# Rare Coexistence of Pemphigus Vulgaris and Eosinophilic Esophagitis: A Report of Two Cases

**DOI:** 10.1002/ccr3.70388

**Published:** 2025-04-01

**Authors:** Joe Khodeir, Paul Ohanian, Sarah Saleh, Karam Karam, Emanuel‐Youssef Dib, Houssein Chebbo, Elias Fiani

**Affiliations:** ^1^ Department of Dermatology Saint Georges Hospital University Medical Center, University of Balamand Beirut Lebanon; ^2^ Department of Family Medicine University of Balamand Beirut Lebanon; ^3^ Department of Internal Medicine University of Balamand Beirut Lebanon; ^4^ Department of Gastroenterology University of Balamand Beirut Lebanon

**Keywords:** bullous disease, eosinophilic esophagitis, esophageal disease in pemphigus, pemphigus vulgaris

## Abstract

Dysphagia in pemphigus vulgaris (PV) patients should not be attributed solely to PV. Eosinophilic esophagitis (EoE) can coexist and requires endoscopic evaluation and biopsy for accurate diagnosis. Both conditions may share underlying immune mechanisms, emphasizing the importance of precise diagnosis and targeted treatment.

## 1 Introduction

Pemphigus vulgaris (PV) and eosinophilic esophagitis (EoE) are autoimmune and immune‐mediated disorders, respectively, that impact distinct organ systems [1, 2]. PV is characterized by the presence of autoantibodies targeting desmogleins, leading to skin and mucosal blistering [1]. EoE, on the other hand, involves eosinophilic infiltration of the esophageal epithelium, typically associated with allergic conditions [2]. The concurrent presentation of PV and EoE is rare and poses significant diagnostic challenges [3]. This report examines two cases where patients experienced both conditions, highlighting the need for careful differentiation between esophageal involvement by PV and EoE. These cases illustrate the importance of a thorough diagnostic workup, including endoscopy and biopsy, to ensure accurate diagnosis and effective treatment.

### 1.1 Case 1

#### 1.1.1 Case History and Examination

**FIGURE 1:**
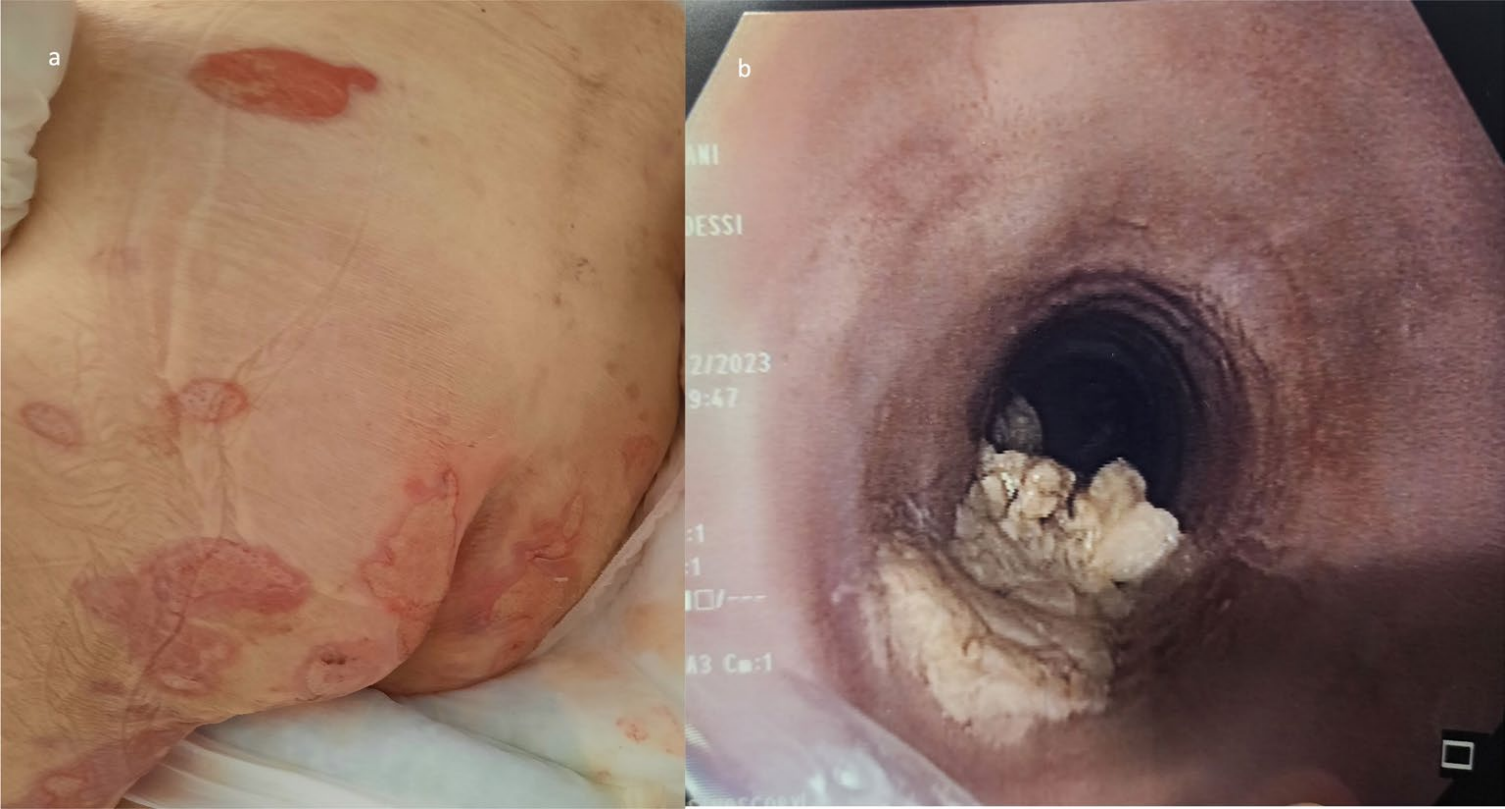
(a) Flaccid bullae and erosions on lower legs and buttocks. (b) Esophagogastroduodenoscopy revealing impacted food bolus, esophageal rings and furrows.

A 47‐year‐old male patient presented to the emergency department with difficulty swallowing after eating a piece of steak. He reported several episodes of food getting “stuck” in his esophagus. This dysphagia was not associated with any other alarming signs, including weight loss or fever. Four months prior, he was diagnosed with PV after presenting with painful blisters and erosions all over his body following painful oral erosions. At that time, physical examination revealed flaccid bullae and erosions all over his body (Figure 1a), with no genital or ocular lesions, no lymphadenopathy, and no associated systemic signs. PV was confirmed by a skin biopsy revealing suprabasal epidermal acantholysis and direct immunofluorescence showing intercellular deposits of C3 and IgG in the epidermis. He had been treated with oral prednisone at a dosage of 1 mg/kg/day, which was tapered over 4 weeks, leading to full remission with no relapse on follow‐up 3 months later.

#### 1.1.2 Differential Diagnosis, Investigations, and Treatment

The differential diagnosis for the patient's current presentation included a relapse of PV, specifically esophageal mucosal involvement by PV. Another possibility on the differential was EoE. An urgent upper endoscopy revealed a meat bolus lodged in the mid‐esophagus along with esophageal rings and furrows (Figure 1b). Multiple biopsies were taken to rule out esophageal involvement by PV or an EoE. Histology showed 20 eosinophils per high‐power field in the squamous mucosa, with foci of eosinophilic micro‐abscesses (Figure 3a) and no sign of PV such as acantholysis in the lower spinous cell layers. Direct immunofluorescence exam was also negative, excluding a PV. Once the diagnosis of EoE was confimed the patient was restarted on prednisone at a dosage of 0.5 mg/kg per day and maintained on oral proton pump inhibitors (PPI). Three weeks after resuming prednisone, the patient's esophageal symptoms subsided. At a six‐month follow‐up, there were no signs of relapse of either EoE or PV.

### 1.2 Case 2

#### 1.2.1 Case History and Examination

**FIGURE 2:**
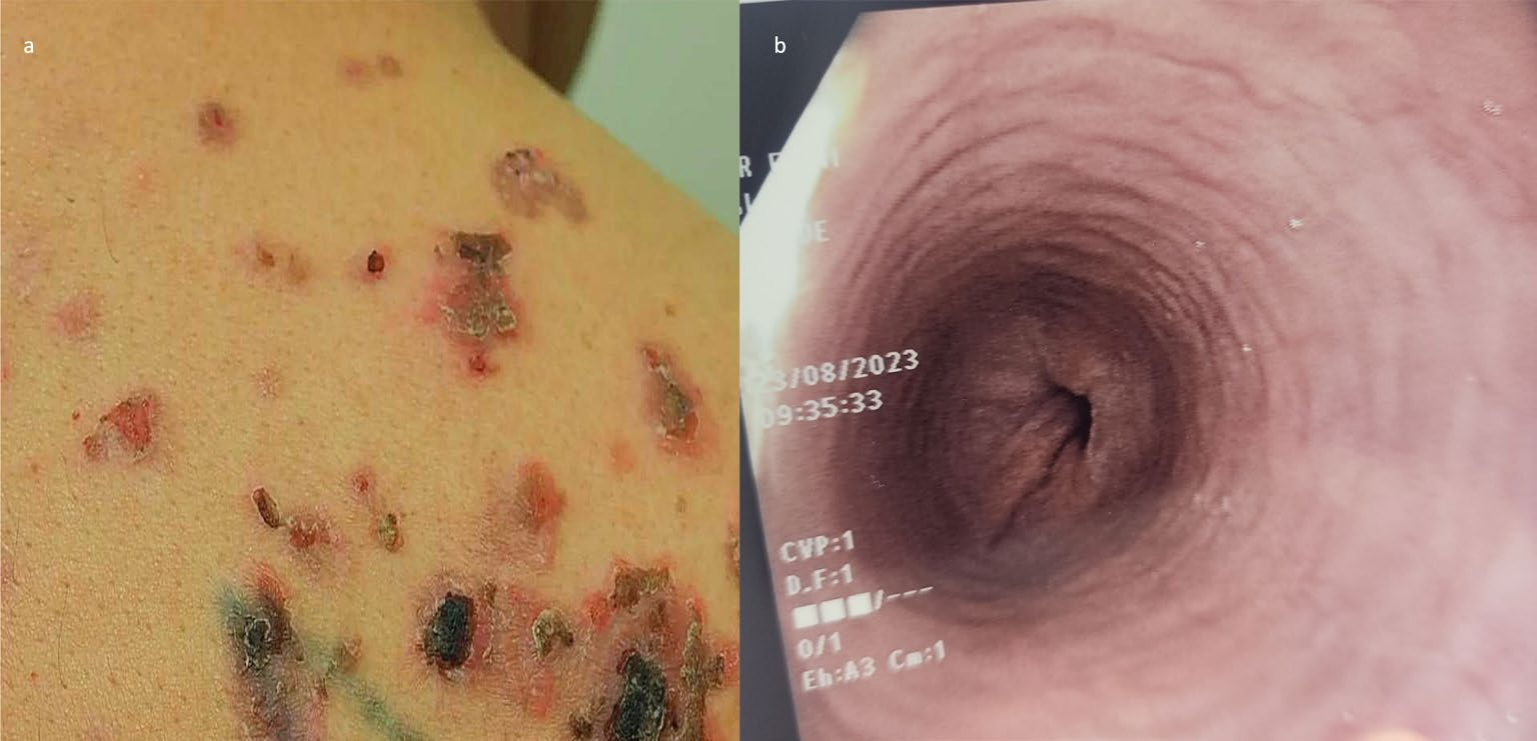
(a) Bullae and erosions on the back of the patient. (b) Esophagogastroduodenoscopy revealing a web like structure in the distal esophagus.

A 55‐year‐old male with no significant past medical history presented with a two‐month history of difficulty swallowing solid food without any systemic alarming signs. Endoscopy revealed a stenotic gastroesophageal junction, with furrows and a web‐like structure observed above it (Figure 2b). Biopsies taken from these areas demonstrated numerous eosinophils, consistent with a diagnosis of eosinophilic esophagitis (EoE). The patient was started on oral steroids at a dosage of 1 mg/kg per day for 4 weeks, followed by a tapering regimen, which led to significant improvement in his dysphagia. He remained asymptomatic for 6 months. After this period, the patient sought a dermatology consultation for an acute rash. He presented with flaccid bullae on his chest, back, buttocks, lower legs, and anterior oral mucosa (Figure 2a).

#### 1.2.2 Differential Diagnosis, Investigations, and Treatment

**FIGURE 3:**
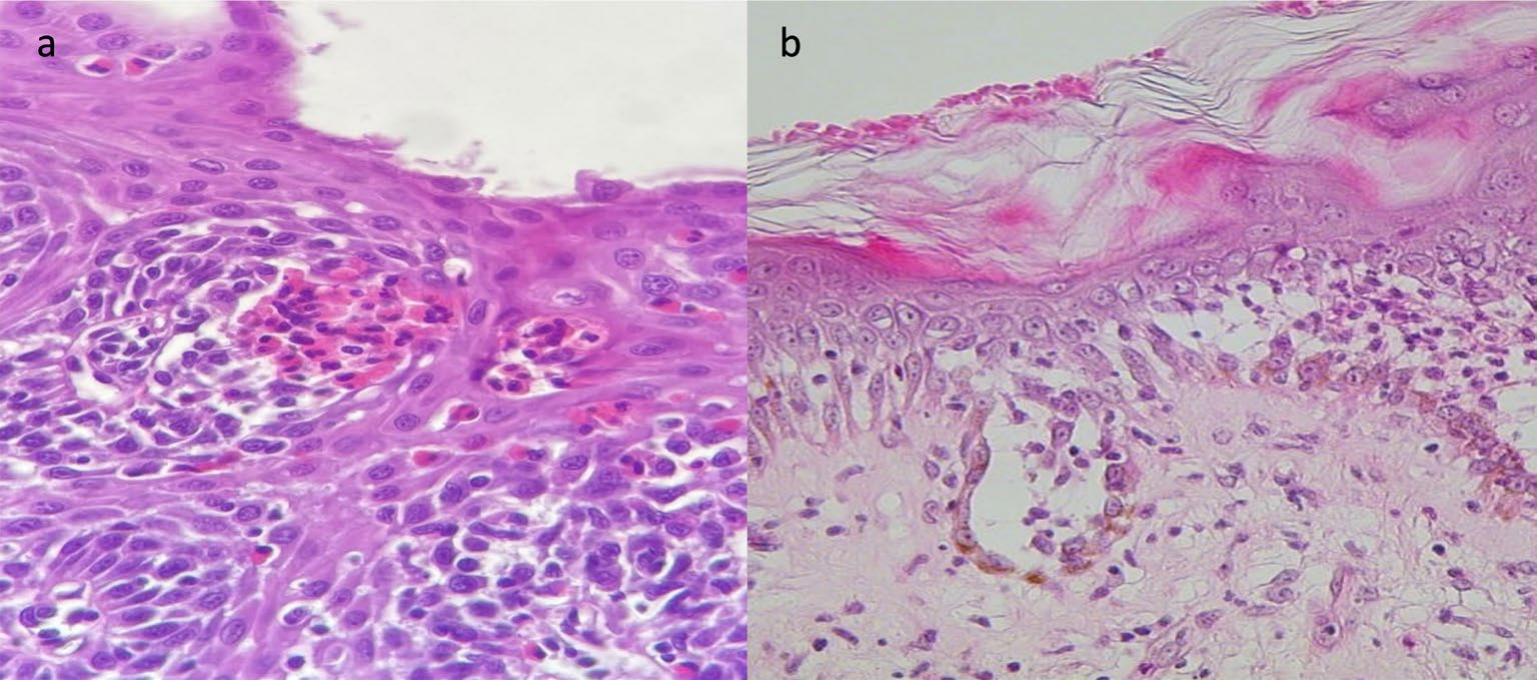
(a) Esophageal biopsy showing numerous eosinophilic infiltrates in the mucosa along with an eosinophilic micro abscess (hematoxylin and eosin stain HE ×100). (b) Skin biopsy revealing suprabasal epidermal acantholysis (hematoxylin and eosin stain HE ×100).

The differential diagnosis for the acute rash included PV and other autoimmune blistering diseases. A skin biopsy and direct immunofluorescence (DIF) confirmed the diagnosis of PV, showing suprabasal epidermal acantholysis (Figure 3b) and intercellular deposits of C3 and IgG in the epidermis. The patient was initially treated with oral prednisone at a dosage of 1 mg/kg per day for 4 weeks, followed by a tapering regimen. He was then switched to azathioprine at a dosage of 2 mg/kg per day due to relapse after stopping the systemic steroid. Ten months later, he experienced a relapse in both EoE and PV upon stopping azathioprine. A decision was made to start him on dupilumab for his EoE, beginning with a 600 mg loading dose, followed by 300 mg every 2 weeks. This treatment resulted in full remission of both diseases, and the patient was kept on it as a chronic therapy.

## 2 Discussion

The coexistence of PV and EoE in these two cases presents a unique clinical scenario, with only one case report in the medical literature presenting a similar association [3], highlighting the complexity and interplay of autoimmune diseases. This association might be underrecognized due to the direct assumption that every patient with PV who presents with esophageal symptoms, such as difficulties in alimentation, is due to the involvement of the esophageal mucosa by the disease itself, and not considering an alternative diagnosis such as EoE. The physician might not search for another pathology due to the response of both diseases to the same therapies such as systemic cortico‐therapy [2, 3, 4]. PV is an autoimmune blistering disorder primarily affecting the skin and mucous membranes, while EoE is a chronic immune‐mediated esophageal condition characterized by eosinophilic infiltration and esophageal dysfunction, predominantly associated with allergic conditions [1, 2]. Both cases underscore the necessity of a thorough and multidisciplinary diagnostic approach when dealing with patients presenting with new or unusual symptoms, particularly in those with known autoimmune conditions. In Case 1, the patient developed dysphagia and esophageal impaction, which necessitated a biopsy to distinguish between esophageal involvement by PV and EoE. In Case 2, the patient initially presented with symptoms of EoE, which were managed successfully, only to later develop PV. The diagnosis of EoE in both cases was confirmed by endoscopic findings and biopsies demonstrating significant eosinophilic infiltration. The presence of both conditions in the same patients suggests a possible shared immunopathogenic mechanism, although the exact pathophysiology remains unclear [3]. The simultaneous occurrence of both conditions is exceptional in the medical literature, and dysphagia in PV patients is often empirically attributed to mucosal involvement of the esophagus by PV [4, 5]. The shared pathogenesis of PV and EoE might be linked to the involvement of desmoglein (DSG) proteins, mainly DSG‐1 [6]. Our findings are consistent with a previously reported case by Gue et al. in 2017 documenting a 13‐year‐old boy with PV and EoE, emphasizing the possible dysregulation of DSG 1 as a common etiologic factor in both conditions [3]. Eosinophils play a central role in EoE and have also been implicated in the pathogenesis of PV, suggesting a shared pathogenic mechanism [1, 2]. The significant improvement in both EoE and PV symptoms in our patient treated with dupilumab, a monoclonal antibody targeting the IL‐4 receptor alpha that is FDA‐approved for the treatment of EoE and used off‐label for recalcitrant PV cases [7, 8, 9], suggests that Th2‐mediated pathways might be a common underlying mechanism in both diseases [10, 11, 12].

## 3 Conclusion

The coexistence of PV and eosinophilic esophagitis EoE in these cases highlights the complex interplay between autoimmune diseases and underscores the necessity of a thorough and multidisciplinary diagnostic approach. Our findings demonstrate that EoE should be considered in PV patients presenting with dysphagia, as it is essential not to attribute such symptoms solely to esophageal involvement by PV. Thus we recommend routine biopsies for PV patients who present with dysphagia. The coexistence of both diseases might be due to a shared underlying mechanism or a coincidental occurrence and cannot be concluded from two cases only. Further case accumulation is needed to clarify this relationship. The significant improvement observed in both conditions with the use of dupilumab suggests a shared Th2‐mediated pathway, opening new avenues for therapeutic intervention. Further research is needed to elucidate the underlying mechanisms linking PV and EoE.

## Author Contributions

**Joe Khodeir:** conceptualization, data curation, investigation, methodology, validation, writing – original draft, writing – review and editing. **Paul Ohanian:** data curation, investigation, methodology, writing – original draft. **Karam Karam:** investigation, methodology, project administration. **Sarah Saleh:** resources, writing – original draft. **Houssein Chebbo:** writing – original draft. **Emanuel‐Youssef Dib:** resources, writing – original draft. **Elias Fiani:** supervision, validation.

## Consent

Written informed consent was taken from each patient to publish their data and figures.

## Conflicts of Interest

The authors declare no conflicts of interest.

## Data Availability

The data used to support the findings of this study are included within the article. I, Joe Khodeir, have full access to all the data in the study and take responsibility for the integrity of the data and the accuracy of the data analysis.
